# Impact of Supportive Leadership During Covid-19 on Nurses' Well-Being: The Mediating Role of Psychological Capital

**DOI:** 10.3389/fpsyg.2021.695091

**Published:** 2021-09-30

**Authors:** Um-e-Rubbab, Tahir Farid, Sadaf Iqbal, Imran Saeed, Shahid Irfan, Tanvir Akhtar

**Affiliations:** ^1^Department of Business Administration, Fatima Jinnah Women University, Rawalpindi, Pakistan; ^2^Department of Psychology and Behavioral Sciences, Zhejiang University, Hangzhou, China; ^3^Department of Psychology, Abdul Wali Khan University Mardan, Mardan, Pakistan; ^4^Institute of Business and Management Science, The University of Agriculture, Peshawar, Pakistan; ^5^Department of Psychology, Foundation University, Islamabad, Pakistan

**Keywords:** supportive leadership, psychological capital, psychological well-being, social well-being, nurses, healthcare sector

## Abstract

The corona virus disease (Covid-19) has significantly affected the social, physical, and psychological health of workers, specifically the nurses working in the healthcare sectors. Studies have been conducted on the impact of Covid-19 on employees' well-being, organizational structure, and job design; however, limited studies have been conducted focusing on the impact of leadership on employee's well-being during the Covid-19 pandemic. Drawing on job demands resources model and social exchange theory, we examined the impact of supportive leadership on employees' physical, social, and psychological well-being during the Covid-19 pandemic. In addition, we examined the mediating role of psychological capital in examining the relationship between supportive leadership and employees' physical, social, and psychological well-being. Based on three wave time-lagged design, the data were collected from 214 nurses' linear regression analysis and Hayes Process for mediation to test the proposed hypothesis. As hypothesized, supportive leadership predicted employees' physical, social, and psychological well-being. In addition, psychological capital mediated the relationship between supportive leadership and employees' physical, social, and psychological well-being. Implications for research, theory, and practice are discussed.

## Introduction

Corona virus (Covid-19) is a new disease and is considered to be a very serious threat to all human beings. It was first started in December 2019 from Wuhan Province of China and quickly spread across the globe (World Health Organization, 2020). It created a huge psychological and mental pressure that increased very quickly among people and paramedical staff across the globe (Irshad et al., 2020). Scientists and medical professionals were caught unaware and did not know how to control and treat Covid-19 patients (Prompetchara et al., 2020). By the end of 2020, there were no successful vaccines or drugs yet for treatment of the virus (Ahmed et al., 2020; Prompetchara et al., 2020; Sanders et al., 2020); only a few vaccines were in experimental stages. Since the outbreak of this pandemic, researchers have been trying to investigate the antecedents that can result in safe workplace behaviors and protect nurses' well-being. Limited research has been conducted in this situation and how to focus on the relationship between supervisors and subordinates (Zhao et al., 2020). A study conducted by McGilton et al. (2009) suggested that the supervisor-subordinate relationship can be improved by focusing on effective supervisory behavior, which in turn can help to build their psychological resources.

Due to Covid-19 almost every sector of the world has been disturbed, and specifically the healthcare sector was the most affected sector (Zhao et al., 2020). Paramedic staff specifically and nurses around the globe are facing life-threatening job-related risks and stayed isolated due to the transmissible nature of the deadly virus (Mo et al., 2020). Nurses in Pakistan are reporting symptoms of depression and anxiety affecting their physical and mental well-being (Dawn News, 2020). The nurses' safety and psycho-social well-being is essential to enhance their capacity to take care of patients suffering from this disease. It has been noted that leadership styles can play a pivotal role in responding to such situations of crises by uplifting employees' well-being (Dirani et al., 2020). Considering the challenging conditions caused by the pandemic, this study aims to identify how supportive leadership can affect nurses' well-being at work. In particular, from the perspective of supportive leadership, we discuss how psychological capital is developed and the mechanism through which it mediates between supportive leadership and nurses' well-being.

Thus, the main aim of this study is to add knowledge to the existing literature by examining two important issues. First, the literature on supportive leadership in the healthcare sector context during such crises is limited. We examine the role of supportive leadership behaviors in influencing nurses' well-being during the Covid-19 pandemic. Organizational factors, i.e., support from others, work climate, and support of supervisor, may contribute to the well-being of healthcare workers (Joiner et al., 2004; Arnetz and Blomkvist, 2007; Lohela et al., 2009; Irshad et al., 2020). Supportive leadership is defined as those attitudes, actions, behaviors, and communications by supervisors that help workers by enabling them to working effectively, productively, and appropriately (Muller et al., 2009). Nurses' well-being including psychological, social, and physical well-being is the proposed outcome of supportive leadership in this study. This is consistent with the study of Dodge et al. (2012) that well-being consists of three types of resources: psychological, physical, and social.

Second, focusing on a job demands resources model (Bakker and Demerouti, 2008; Bakker, 2011), we build a conceptual framework and investigate the mediating effect of psychological capital. In light of positive psychology, psychological capital is one the personality constructs that refers to “an individual positive psychological state of development” (Luthans et al., 2007a). The individuals with high psychological capitals have the ability to overcome the pandemic related crises and invest more in their workplace with esteemed dedication. Therefore, this study proposes that supportive leadership behavior enhance employees' well-being through psychological capital.

Conducting research on the supportive leadership and nurses well-being during this Covid-19 pandemic is important in the context of the healthcare sector in Pakistan because most of the studies related to these topics have been conducted in the Western culture which is totally different from Pakistani culture. Hence, this would be a new addition to the existing literature by examining the aforementioned relationship in the collectivistic culture of Pakistan. By examining the process through which psychological capital transmits the effect of supportive leadership on nurses' well-being, this study extends the supportive leadership and psychological capital literature in a new distinct direction. Moreover, the findings of this study will enrich relative research on the role of supportive leadership and nurses' well-being and how these factors can improve the health sector. Although managers and their subordinates both are influenced by surroundings, supervisors have greater potential to restructure it and managers by interacting with environment and can influence the health of subordinates (Nyberg et al., 2005).

The next section presents the literature review. The relationships between variables are clearly linked with prior literature justifications. Hypotheses are developed on the basis of past literature.

## Literature Review

### Theory and Hypotheses Development

We used social exchange theory (Blau, 1964) for our framework which is the most noticeable theory in the area of organizational behavior. Social exchange theory approaches the behavior of the leader and subordinate (Gouldner, 1960), and it further examines the idea that the good behavior of the leader has a great impact on the organization (Liborius, 2014).

Social exchange theory exhibits good relationship, trust, and mutual understanding (Li and Liao, 2014). This theory reveals that trusting the employees can bring positive change in the organization and motivate them toward their targeted goals (Gouldner, 1960; Blau, 1964). Close association among the employees and the leader can resolve various issues; by different means of communication they can reach and achieve their common goals (Usman et al., 2021). Based on social exchange theory, we exhibit that when leaders encourage their employees and support them during stressful situations, it helps in the well-being of the employees.

### Supportive Leadership and Nurses' Well-Being

Employees' well-being has been divided into three dimensions which are as follows: psychological, physical, and social well-being (Van De Voorde et al., 2012). Van De Voorde et al. (2012) further elaborated that psychological well-being is related to happiness and subjective experience at work, whereas social capital is related to relationships and quality of interaction between employees or between employees and supervisors. Physical well-being is termed as the health of workers (Chou et al., 2002; Grant, 2007; Van De Voorde et al., 2012) and it is related to those situations which may give rise to strain, psychological distress, physical illness, and burnout among employees (Spector and Jex, 1998; Van De Voorde et al., 2012). Psychological and physical well-being is centered on individuals mainly, and social well-being is focused on interaction with others (Mehra et al., 2016).

Leader behavior that involves expression of concern and emotional support for employees' needs and well-being is known as supportive leadership (House, 1971; Rafferty and Griffin, 2004; Shin et al., 2016). The supportive role of a leader is extremely critical in current Covid-19 situations where conserving and maintaining nurses' well-being can enhance the healthcare conditions. Supportive leadership provides individualized consideration to followers and attends and responds to their personal needs, and it also focuses on provision of social and emotional support to the followers (House, 1981; Rafferty and Griffin, 2004).

Moreover, supportive leadership is manifested in behaviors such as sympathizing to followers, listening to them, and providing care to them (House, 1981). This specific feature, i.e., individualized consideration in term of provision of emotional support to the followers, makes supportive leadership more effective and distinguishes it from transformational leadership which is mainly concerned with organization as a whole (Rafferty and Griffin, 2004). Thus, when nurses consider their leader as supportive toward them and find them listening, sympathizing, and providing emotional support, their well-being is maintained and restored. When nurses have a frustrating day or they are going through turmoil at work, they not only expect their supervisor to be available to them and informed of the situation but also need the supervisor's encouragement and support, and this is considered the supportive behavior of the supervisor (McGilton, 2010).

**Figure 1:**
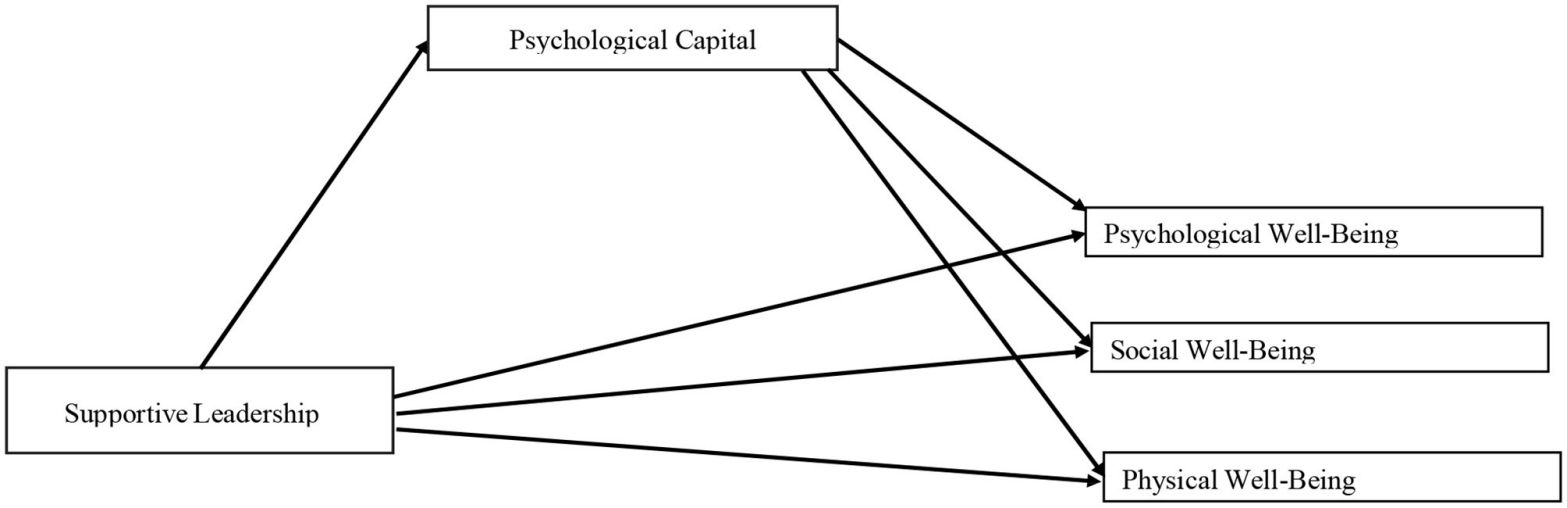
Hypothesized model.

Therefore, we anticipate that there is positive relationship between supportive leadership and nurses' well-being, i.e., emotional, psychological, and physical well-being. When a follower perceives his/her leader to be supportive toward them, he/she engages in supportive behaviors toward others at the workplace too (Shin et al., 2016), and this helps nurses to tackle the emotional and physical demands of work (Figure 1).

H1: Supportive leadership during Covid-19 is positively associated with nurses' physical well-being.
H2: Supportive leadership during Covid-19 is positively associated with nurses' social well-being.
H3: Supportive leadership during Covid-19 is positively associated with nurses' psychological well-being.

### Psychological Capital as Mediator Between Supportive Leadership and Nurses' Well-Being

Psychological capital is defined as a positive psychological state and consists of four dimensions: hope, self-efficacy, resilience, and optimism (Luthans et al., 2007b), and all of these facets are an individual's personal resources. When leaders are supportive, it results in nurses' psychological capital which would further influence the well-being of nurses. Similarly, when leaders are supportive toward nurses by listening, sympathizing, and showing concern to their needs and challenges, it is expected that nurses will also express optimism for future and hope, confidence, and resilience (Li et al., 2018).

According to the job demands resources model (Van Der Heijden et al., 2008; Bakker, 2011), resources an individual possesses (e.g., personal and job resources) can directly influence his/her health and well-being at work. Supportive leadership is a job resource for nurses that helps them to develop their psychological capital (Schaufeli and Taris, 2014), which is a personal resource for nurses.

Job resources and personal resources interact and interrelate with each other, helping to improve the well-being of nurses. Supportive leaders inspire and motivate their followers. According to job design resources model (Bakker, 2011), supportive leadership being a job resource correlates with nurses psychological capital that is a personal resource. An individual's psychological capital may vary depending upon his/her contextual factors that is the support of leader (Luthans et al., 2007b). Nurses with high psychological capital will be more likely to overcome the pandemic-related deteriorating work demands and would invest more to their workplace with esteemed energy and vigor. Supportive leadership has the potential to enhance followers' psychological capacities, i.e., hope, self-efficacy, confidence, and optimism. Employees with higher psychological capital are more tough, optimistic toward work, and full of hope (Luthans et al., 2004). Similarly, workers possessing high resilience can deal with challenges and can manage their emotions and attitude effectively (Ziyae et al., 2015; He et al., 2016). According to psychological resources theory (Gorgievski et al., 2011), support from leaders can energize employees' psychological resources, i.e., psychological capital (confidence, hope, resilience, and optimism), which will help them to improve well-being at work. Taken together, it is predicted that when nurses perceive their leaders to be supportive, it increased their psychological capital, which in turn would lead nurses to improve the well-being of nurses. So, the following mediating hypotheses are proposed:

H4: Psychological capital mediates the relationship between supportive leadership and nurses' physical well-being.
H5: Psychological capital mediates the relationship between supportive leadership and nurses' social well-being.
H6: Psychological capital mediates the relationship between supportive leadership and nurses' psychological well-being.

## Materials and Methods

### Sample and Procedure

The current study is quantitative in nature. Directly visiting hospitals was risky and physical contact was not possible with nurses due to Covid-19 restrictions. All nurses were approached online. The current study followed CHEERIES checklist and DTROBE checklist for electronic surveys and time lagged studies, respectively. Our study was approved by institutional review board and followed all ethical guidelines. Data for all variables were obtained from nurses themselves; thus, it was self-reported, which is not free of common method biasness. Data for all three measures were collected in three time lags. Each time lag consisted of a minimum gap of 10 days, and this is consistent with the recommendations of Podsakoff et al. (2012). There are multiple other studies which have employed a time lagged approach to collect data for minimizing common method bias (Irshad et al., 2020; Majeed et al., 2020).

Pakistan reported its first Covid-19 case in the end of February 2020 (Malik et al., 2020; Shahid et al., 2020) and numbers of cases were increasing throughout Pakistan during the data collection process. The data collection process began during the second wave of Covid-19 in Pakistan on November 15, 2020, and ended on December 5, 2020. The country saw smart lockdowns in various major cities, and the deadly virus was spreading, victimizing, and killing people during the data collection process. Hospitals were witnessing again a sudden surge in patients. The second wave of Covid-19 was appearing with novel and more dangerous life taking symptoms, creating more challenges for the healthcare system. Non-probability convenience sampling techniques were used for data collection in the current study. Authors collected e-mail addresses of nursing staff through personal contacts and hospitals websites where possible. Data were collected from 17 public hospitals. Informed consent was obtained from nurses before participation and they were not forced to respond at any stage during the data collection process. Respondents were free to quit the process and refuse to be involved in the data collection procedure. Informed consent was clearly mentioned before starting questionnaires that their participation is entirely voluntary. They were assured that their confidentiality would be kept intact and their names will not be shared. They were also briefed that results of the study would also be shared with them if they were interested. We created Google forms to get their responses on measures. These Google forms were emailed to potential nurses along with the explicitly mentioned purpose of the study and informed consent. Nurses were able to review all the given responses before submission. Only one response was received against one email address as per CHEERIES checklist for conducting online surveys, thus the setting on Google forms was customized to one response from one nurse at a time, i.e., time 1, time 2, and time 3. Surveys were very brief and concise and thus required very little time to read and complete.

All three scales were presented one by one and in a sequence with time lags. Nurses were asked to provide information on demographics and supportive leadership at time 1. Informed consent and purpose of research was also explained in the first section presented at time 1. Required information on demographics included age, tenure, and education. At time 2, nurses' response on psychological capital was obtained with a time lag of 10 days. At time 3, nurses were asked to provide their response on psychological well-being, social well-being, and physical well-being. Anonymity of respondents was fully assured and maintained. A total of 322 questionnaires were e-mailed at time 1, out of which 270 returned with a response rate of 0.84%. At time 2, only those nurses were contacted who responded to time 1 e-mails. At time 2, 251 nurses responded out of 270. At time 3, only 251 nurses were contacted for response on physical, social, and psychological well-being. Final response rate was 214 nurses. These 214 nurses' response was included only for data analysis. Sample adequacy was then assessed through G^*^Power (version 3.1.9.4) (Faul et al., 2009). For small effect size, default value is.02 according to the G^*^Power version (3.1.9.4), for medium effect size it is 0.15, and for large effect size it is 0.35. These same default parameters were used (Faul et al., 2009). F- Test was used to calculate the sample size and linear multiple regression was employed.

Out of the total 214 nurses, 42% were male nurses and 58% were female. A total of 24% of the nurses were between age group 21–30 years old, 23% nurses were between age group 31–40 years old, and 31% nurses were between age group 41–50 years old while 21% were aged 51 and above. In addition, 48% had experience <5 years, 28% had experience of 6–10 years, and 24% had experience of more than 10 years (see Table 1, Sample Characteristics).

**Table 1:** Confirmatory factor analysis and alternative models.

**Model**	**χ^**2**^**	**df**	**χ^**2**^/df**	**CFI**	**TLI**	**IFI**	**GFI**	**RMSEA**
**Hypothesized five factor model (SL, PsyCap, Physical, Social, and Psychological Well-Being)**	**1,828**	**1,474**	**1.24**	**0.94**	**0.93**	**0.94**	**0.78**	**0.034**
Hypothesized three DVs, three factor model (Psychological Well-Being, Social Well-Being, Physical Well-Being)	138	116	1.19	0.98	0.98	0.98	0.92	0.30
Alternate models: Three DVs two factor model (Psychological Well-Being + Social Well-Being, Physical Well-Being	263	118	2.23	0.91	0.89	0.91	0.84	0.076
Three DVs two factor model (Social Well-Being + Physical Well-Being, Psychological Well-Being)	280	118	2.38	0.89	0.88	0.90	0.83	0.08
Three DVs, two factor model(Social Well-Being, Physical Well-Being + Psychological Well-Being)	263	118	2.23	0.91	0.89	0.91	0.84	0.07
Three DVs, one factor model (Psychological Well-Being, Social Well-Being, Physical Well-Being)	384	119	3.22	0.83	0.81	0.83	0.78	0.10
Hypothesized PsyCap one factor model (hope + resilience + optimism + self-efficacy)	476	252	1.89	0.90	0.89	0.90	0.84	0.06

*SL, Supportive Leadership; PsyCap, Psychological capital*.

### Instruments

All the questionnaires used in this study were adapted. Questionnaires were distributed in the English language. English is easily understood and mainly used within hospitals. Many earlier studies have also used the English language scales for data collection purposes from hospitals (Irshad et al., 2020; Majeed et al., 2020). The data were collected from the same respondents which might cause common method bias. To rule out the possibility of common method bias, we collected the data in multiple time lags. In addition, when all items of the survey instrument were loaded on a single factor, the Harman's single factor reflects the estimated shared variance of 31.08%, which is far less than the recommended threshold value of 50%. Thus the findings of our study are not significantly influenced by common method variance.

### Supportive Leadership

Supportive leadership was measured with a 15-item scale developed by McGilton (2010). Responses were measured on a 5-point Likert scale ranging from 1 for *rarely* and 5 for *very often*. The wordings of the items were modified to assess the impact of supportive leadership during Covid-19. Sample items include “During Covid-19 pandemic, my supervisor recognizes my ability to deliver quality care” and “During Covid 19 pandemic, my supervisor encourages me even in difficult situations.” The Cronbach alpha for this study was 0.94. Other studies have also used a similar scale for testing supportive leadership style (Samuel et al., 2018; Rodríguez-Monforte et al., 2021).

### Psychological capital

Psychological capital was measured using the 24-item scale developed by Luthans et al. (2007). Psychological capital was measured as a state for this study. PCQ is a self-administered questionnaire which consists of four sub scales (hope, resilience, self-efficacy, and optimism). Each subscale consists of six items. Items 1–6 are related to self-efficacy, items 7–12 are related to hope, items 13–18 are related to resilience, and items 19–24 are related to optimism. Nurses were asked to respond on the statements based on a 6-point Likert scale with 1 for *strongly disagree* and 5 for *strongly agree*. Sample items include “I am optimistic about what will happen to me in the future as it pertains to work” and “I usually manage difficulties one way or another at work.” Cronbach alpha of psychological capital in this study was 0.94. Other scholars have also used 24-item scales for measuring psychological capital of employees (Raja et al., 2020; Purwanto et al., 2021).

### Nurses' Well-Being

Nurses' well-being was measured using the scale of Van Veldhoven and Broersen (2003). It is a general well-being scale that consists of three categories. Psychological well-being consists of 6-item scale. Cronbach alpha for this study was 0.84. Nurses' response was measured on a 5-point Likert scale with 1 for *strongly disagree* and 5 for *strongly agree*. Sample items include “I continually have to overcome resistance in order to do my work” and “I enjoy my work.” Physical well-being consists of 5 items. Cronbach alpha for this study was 0.84. Nurses response was measured on a 5-point Likert scale with 1 for *strongly disagree* and 5 for *strongly agree*. Sample item includes “I feel fit during work” and “I am very energetic at work.” Social well-being was measured through a 6-items subscale. Cronbach alpha for this study was 0.84. Nurses' response was measured on a 5-point Likert scale with 1 for *strongly disagree* and 5 for *strongly agree*. Sample items include “Can you count on your colleagues when you encounter difficulties in your work? “and “If necessary, can you ask your colleagues for help?” Other studies have also used a similar scale for measuring employees' well-being (Verbraak, 2014).

### Data Analysis

For data analysis, Statistical Package for Social Sciences SPSS version 21 and AMOS version 21 was used for this study. Health of the data was checked and we confirmed that data had no issues of missing values and multicollinearity. All correlations were under the cut-off value of 0.70 and for all the variables, variance inflation factor was below the cut-off score of 10 and tolerance value was also above the threshold value, i.e., 0.2 (Myers and Myers, 1990; Menard, 2000). Data fulfilled all the basic assumptions of regression.

### Measurement Model

AMOS was used to run confirmatory factor analysis. Maximum Likelihood was used in AMOS for estimation of parameters as it is recommended for Likert scales (Bai and Li, 2016). Convergent validity was checked for the study variables through factor loadings, and all factor loadings were more than 0.4, thus reflecting that items loaded on their own respective latent factor. A five-factor CFA was conducted to assess the discriminant validity of the study variables. For CFA, values of model fit indices were examined including Chi square, degree of freedom, the root mean square error of approximation, comparative fit index, Tucker Lewis index, and incremental fit index. To check the model fitness, various CFA tests were performed. Three factor model loading with alternate dependent variables were checked. One factor model was also checked by loading all items to a single factor. Comparing the results of all models, the proposed five factor model yielded better fit indices χ^2^ = 1,828, df = 1,474, χ^2^/df = 1.24, *p* <05, CFI = 0.94, TLI = 0.93, IFI = 0.94, RMSEA = 0.034. The values of our five factor model were in the acceptable range (Hair et al., 2014). The values of alternate models showed poorer fit indices than our proposed five factor model. The discriminant validity of our proposed five factor model shows that respondent nurses were able to distinguish the variables.

The overall five factor model yielded good convergent and discriminant validity. For discriminant validity, the average variance extracted (AVE) score for all variables were found less than maximum shared variance (MSV). Further, the loadings of all items on their respective factors were higher than.60, proving the convergent validity of the scales (see Table 2).

**Table 2:** Means, standard deviation, reliabilities, and correlations.

**S.N**	**Variable**	**AVE**	**MSV**	**M**	**S.D**	**1**	**2**	**3**	**4**	**5**	**6**	**7**	**8**	**9**
1	Gender													
2	Age					−0.03								
3	Edu					−0.16*	0.18*							
4	Exp					0.08	0.63**	0.06						
5	SL	0.53	0.24	3.32	0.76	0.03	0.01	−0.03	−0.05	**(0.94)**				
6	P Cap	0.52	0.21	3.32	0.77	−0.01	0.09	0.00	0.08	0.42**	**(0.94)**			
7	Psy Well	0.54	0.19	3.35	0.80	−0.05	−0.05	−0.04	−0.03	0.43**	0.49**	**(0.84)**		
8	Soc Well	0.54	0.53	3.31	0.94	−0.02	0.02	−0.07	−0.05	0.44**	0.41**	0.63**	**(0.84)**	
9	Ph Well	0.52	0.47	3.29	0.89	−0.08	0.10	0.01	0.01	0.37**	0.41**	0.58**	0.59**	**(0.84)**

*SL, Supportive leadership; P Cap, Psychological Capital; Ph Well, Physical well–being; Soc Well, Social well–being; Psy Well, Psychological well–being; Exp, Experience; Edu, Education; S.D, Standard Deviation. Reliabilities are given in bold parentheses. AVE = Average Variance Extracted. MSV, Maximum Shared Variance; AVE, Average Variance Extracted; MSV, Maximum Shared Variance*.

Results of CFA are given in Table 1.

### Correlation Analysis

**Figure 2:**
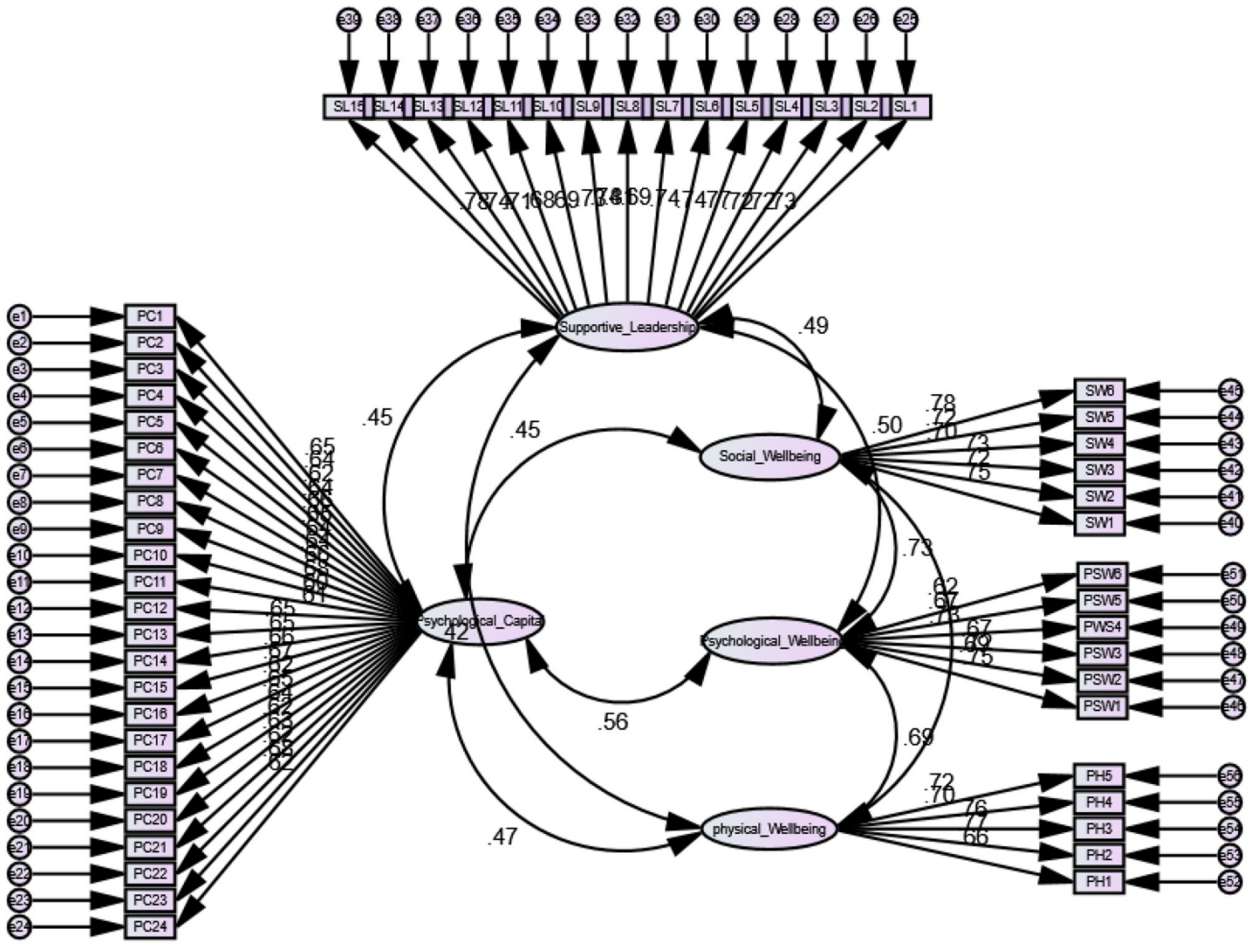
Measurement model.

**Figure 3:**
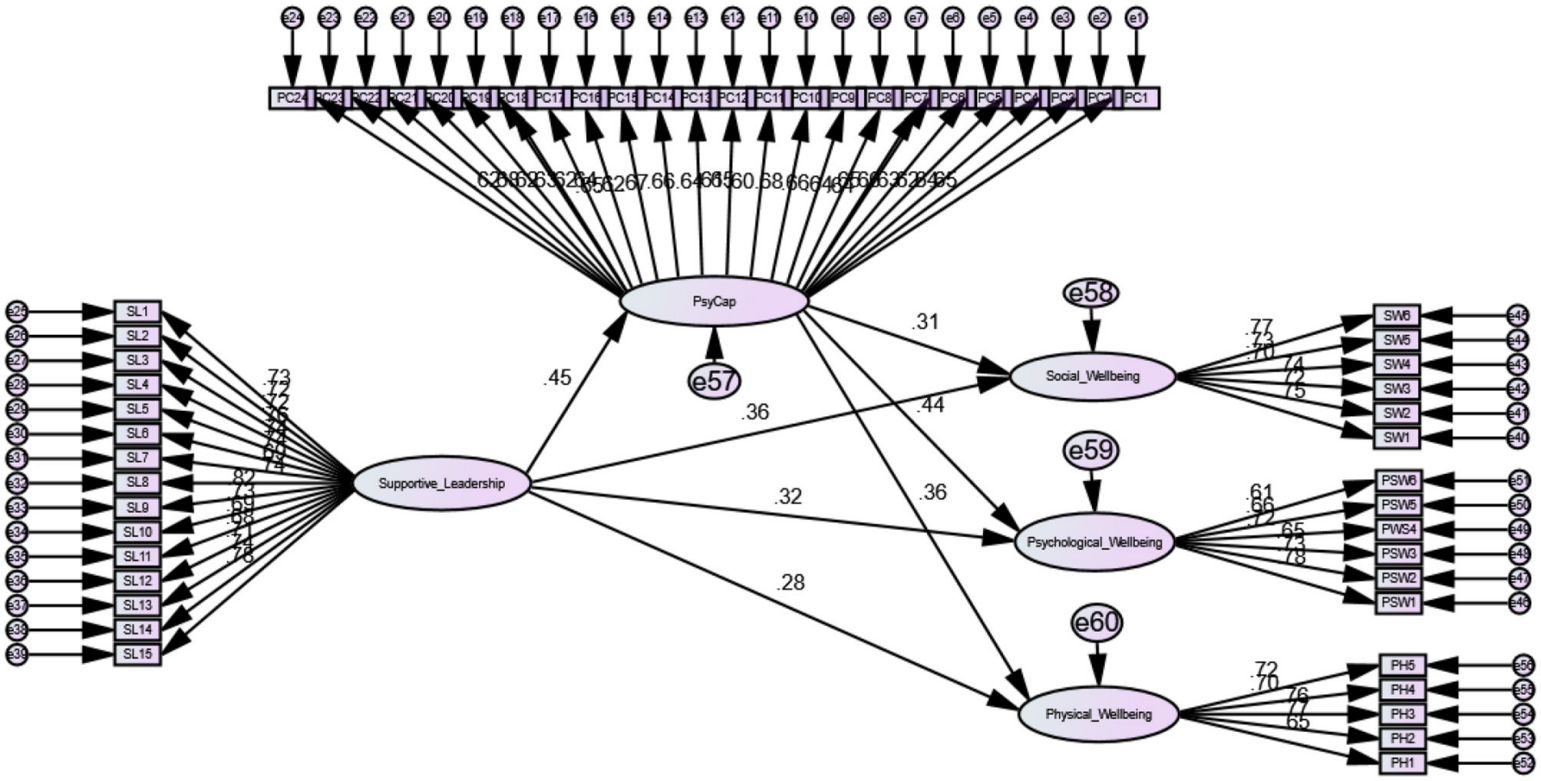
Structural model.

Analysis of variance was performed to rule out the impact of demographics, i.e., age, gender, experience, and education level on study variables. Results of ANOVA showed that all the demographic variables used in the study were non-significant to study variables. All the demographics were not included during SEM analysis. Correlation analysis was performed to assess the correlation between study variables. ANOVA and correlation analysis was performed using SPSS. Results of correlation analysis are shown in Table 2. Supportive leadership during Covid-19 is significantly correlated with nurses' psychological capital (*r* = 0.42^**^, *p* < 0.01), nurses' physical well-being (*r* = 0.37^**^, *p* < 0.01), nurses' social well-being (*r* = 0.44^**^, *p* < 0.01), and nurses' psychological well-being (*r* = 0.43^**^, *p* < 0.01). Nurses' psychological capital is also significantly correlated with nurses' physical well-being (*r* = 0.41^**^, *p* < 0.01), nurses' social well-being (*r* = 0.41^**^, *p* < 0.01), and nurses' psychological well-being (*r* = 0.49^**^, *p* < 0.01) (see Figures 2, 3).

### Structural Equation Modeling

Table 3 provides the results for direct and mediation hypothesis. SEM was used in AMOS to test the mediating hypothesis. Confidence interval was set to 95% and bootstrapping of samples was fixed to 5,000. In line with our study's first hypothesis that supportive leadership during Covid-19 was significantly and positively related to nurses physical well-being, results were found to be significant and positive (β = 0.27, *p* < 0.01), and thus H1 was supported. Our second hypothesis was that supportive leadership during Covid-19 is significantly related to nurses' social well-being. Results were found to be significant and positive (β = 0.36, *p* < 0.01), and thus H2 was supported. Our third hypothesis was that supportive leadership during Covid-19 is significantly related to nurses' psychological well-being. Results were found to be significant and positive (β = 0.32, *p* < 0.01), and thus H3 was also supported.

**Table 3:** Structural equation modeling.

	**Unstandardized β**	**SE**	**95%LLCI**	**95%ULCI**
SL → Physical Well-being	0.28	0.087	0.12	0.45
SL → Social Well-being	0.36	0.083	0.19	0.52
SL → Psychological Well-being	0.32	0.092	0.14	0.51
**Indirect effect**			
SL → PsyCap → Physical Well-being	0.16	0.037	0.09	0.24
SL → Psycap → Social Well-being	0.14	0.036	0.06	0.21
SL → Psycap → Psychological Well-being	0.20	0.043	0.11	0.28

*SL, Supportive leadership; PsyCap, Psychological Capital; SE, Standard Error; LLCI, Lower limit confidence interval; ULCI, Upper limit confidence interval*.

Our fourth hypothesis was that nurses' psychological capital mediates between supportive leadership during Covid-19 and nurses' physical well-being. Result for indirect effects confirmed the significant mediation of psychological capital (indirect effect = 0.16, 95% CI with LL = 0.09 and UL = 0.24). The lower and upper limits of the 95% confidence interval both contain non-zero values. Hence, H4 is supported.

Our fifth hypothesis was that nurses' psychological capital mediates between supportive leadership during Covid-19 and nurses' social well-being. Result for indirect effects confirmed the significant mediation of psychological capital (indirect effect = 0.13, 95% CI with LL = 0.06 and UL = 0.21). The lower and upper limits of the 95% confidence interval both contain non-zero values. Hence, H5 is supported.

Our sixth hypothesis was that nurses' psychological capital mediates between supportive leadership during Covid-19 and nurses' psychological well-being. Result for indirect effects confirmed the significant mediation of psychological capital (indirect effect = 0.19, 95% CI with LL = 0.11 and UL = 0.28). The lower and upper limits of the 95% confidence interval both contain non-zero values. Hence, H6 is also supported. Table 4 contains a summary of the results for all proposed hypotheses.

**Table 4:** Table summary of hypothesis.

**S.No**	**Hypothesis**	**Status**
1	Supportive leadership during Covid-19 positively impacts nurses' physical well-being.	Supported
2	Supportive leadership during Covid-19 positively impacts nurses' social well-being.	Supported
3	Supportive leadership during Covid-19 positively impacts nurses' psychological well-being.	Supported
4	Nurses' psychological capital mediates the relationship between supportive leadership during Covid-19 and nurses' physical well-being.	Supported
5	Nurses' psychological capital mediates the relationship between supportive leadership during Covid-19 and nurses' social well-being.	Supported
6	Nurses' psychological capital mediates the relationship between supportive leadership during Covid-19 and nurses' psychological well-being.	Supported

## Discussion

Findings of our study confirmed that there is a positive and significant relationship between supportive leadership and nurses' psychological well-being. It is consistent with many other studies that claim that employees' well-being can be maintained through certain leadership styles (Bono et al., 2007; Nielsen et al., 2008; Kelloway et al., 2012). This simply means that nurses' psychological well-being is maintained and restored through supportive behavior, concern, and empathy of supervisor. It is suggested that supervisors at all levels especially in healthcare should be encouraged to use supportive strategies to build psychological well-being. Psychological well-being of nurses is not only important for themselves but also critical for their workplace, patients, and loved ones.

Supportive leadership also helps to sustain and improve physical well-being. Supportive behaviors such as encouragement, appreciation, and availability of supervisor when needed by nurses to respond to their needs help them to lower their distress, burnout, and other psychosomatic issues, thus improving their physical well-being. Similarly, supportive leadership behaviors such as lending the support in time of need, understanding and balancing the staff concerns, and keeping workers updated about upcoming issue fulfills nurses' needs related to social well-being. Nurses receiving positive emotions and positive resources from supervisor spill over the other nurses, patients, and their attendants, and thus a contagion of positivity is developed, enabling nurses to maintain the relationships and breaking the knots with their family and friends because of sustained social well-being. Our findings illustrated that positive emotions of leaders through their empathy, listening, and nurturing behavior developed followers' psychological capital and then spilled in positive emotions among them to improve their well-being.

This study has also investigated that how supportive behavior of supervisor influences nurses' well-being. Existing studies suggested that certain organizational or psychological factors (e.g., self-efficacy, hope, work characteristics) can influence the relationship between leadership and well-being (Bakker and Demerouti, 2007; Nielsen et al., 2008; Tafvelin et al., 2011; Mehra et al., 2016). This study supported their notion and highlighted the role of supportive leadership in building nurses' hope for better future, optimism for work challenges, confidence in themselves, and high resilience for meeting Covid-19 chaos, thus building their psychological capital, which in turn helps them to improve their well-being. So psychological capital of nurses proved to be an effective mediator for the relationship between supportive leadership and nurses' well-being.

### Practical Implications, Limitations, and Future Research Suggestions

The current Covid-19 pandemic will continue to disturb our lives for the upcoming years. Thus, it is very important to enhance nurses' well-being as they are the frontline warriors in controlling this pandemic. Our study provides various implications to the healthcare sector. Nurses' psychological capital and well-being are personal resources which help them to deal and manage the patients and their attendants effectively. These resources can be built and strengthened by provision of a supportive work environment. McGilton (2010) stresses the importance of supportive leadership for nurses' positive attitudes at work. Through supportive leadership, nurses find their supervisor available to them, listening, empathetic, and reliable. It is suggested that leaders should keep their workers aware of changes within the work environment.

Nurses would provide intensive care with more confidence to patients and they can give patients' families and their loved one more hope and resilience if they are respected and valued as a person. They need to be appreciated for their limitless efforts and work they do assigned by their immediate supervisors. This will help nurses to maintain their well-being and help them control their fear of being victimized and blame of killing by patients' attendants. Supportive leadership may help nurses to build their resilience and self-efficacy to not cut the knot with the loved ones and for spreading positivity in times of crises. Effective communication and expression of respect and gratitude are key factors for a supportive workplace environment (McGilton, 2010).

Building supportive connection with employees enhances teamwork, cooperation, and better patient outcomes (Anderson et al., 2005). Findings of the study are also consistent with past studies (Tellis-Nayak, 2007), which stressed building quality relationship between nurses and supervisors for creating a person centered workplace which shapes nurses into a devoted caregiver. Our study's findings stressed that supervisors should be encouraged to use supportive management styles to enhance nurses' well-being.

The current study is not without limitations. First, we only examined the healthcare sector, and it is suggested that research should be extended to other workplace settings to further enrich the understanding of the relationships between the examined variables. Second, the majority of our respondents were females which possibly limits our study findings' generalizability to male employees. Finally, this study used psychological capital as a mediating variable, and several other variables such as workplace thriving, organizational identification, and trust could be used as mediating variables to test their models. Specifically, there are many other important factors apart from those examined in the current study which can be examined in the future.

## Conclusion

Covid-19 is devastating for health sector and influencing nurses' well-being significantly. The World Health Organization (WHO) recently issued a warning about the second wave of Covid-19 and alarmed the world that the dangers associated to this pandemic are not over yet, and healthcare sector staff will have to meet the new challenges associated to this deadly infection. Certain management styles, i.e., supportive leadership, can help healthcare staff to build their psychological resources that is psychological capital (hope, resilience, self-efficacy, and optimism) to face the chaos of a pandemic. It is very important to build and maintain the nurses' psychological resources to restore their emotional, social, and physical well-being, and only then they would be able to take the better care of patients and their loved ones. Supervisors at all levels need to be briefed and trained about the issues, challenges, and difficulties their staff is facing to better know and empathize with staff. Supportive leadership thus can help nurses to build personal psychological resources in a difficult environment. Supportive supervisors may lessen staff's tensions and anxieties associated with Covid-19 by lending an ear to hear and shoulder to rely on.

## Data Availability Statement

The raw data supporting the conclusions of this article will be made available by the authors, without undue reservation.

## Ethics Statement

The studies involving human participants were reviewed and approved by the Ethical Committee, Department of Psychology, Foundation University, Islamabad, Pakistan. The patients/participants provided their written informed consent to participate in this study.

## Author Contributions

U-e-R, SIq, and TF wrote the paper, collected the data, and analyzed it. IS, SIr, and TA reviewed and revised the paper. All authors contributed to the article and approved the submitted version.

## Conflict of Interest

The authors declare that the research was conducted in the absence of any commercial or financial relationships that could be construed as a potential conflict of interest.

## Publisher's Note

All claims expressed in this article are solely those of the authors and do not necessarily represent those of their affiliated organizations, or those of the publisher, the editors and the reviewers. Any product that may be evaluated in this article, or claim that may be made by its manufacturer, is not guaranteed or endorsed by the publisher.
